# Plant-derived compounds for treating autosomal dominant polycystic kidney disease

**DOI:** 10.3389/fneph.2023.1071441

**Published:** 2023-02-03

**Authors:** Jieting Zhang, Jiaxin Chen, Jing Xu, Cheng Xue, Zhiguo Mao

**Affiliations:** ^1^ School of Medicine, Shanghai University, Shanghai, China; ^2^ Division of Nephrology, Shanghai Changzheng Hospital, Second Military Medical University, Shanghai, China

**Keywords:** polycystic kidney disease, plant-derived compounds, herbal medicine, treatment, mechanism

## Abstract

Autosomal dominant polycystic kidney disease (ADPKD), the most common monogenic hereditary kidney disease, is the fourth leading cause of end-stage kidney disease worldwide. In recent years, significant progress has been made in delaying ADPKD progression with different kinds of chemical drugs, such as tolvaptan, rapamycin, and somatostatin. Meanwhile, numerous plant-derived compounds have been investigated for their beneficial effects on slowing ADPKD progression. Among them, saikosaponin-d, *Ganoderma* triterpenes, curcumin, ginkgolide B, steviol, resveratrol, *Sparganum stoloniferum* Buch.-Ham, *Cordyceps sinensis*, triptolide, quercitrin, naringin, cardamonin, gambogic acid, and olive leaf extract have been found to retard renal cyst development by inhibiting cell proliferation or promoting cell apoptosis in renal cyst-lining epithelial cells. Metformin, a synthesized compound derived from French lilac or goat’s rue (*Galega officinalis*), has been proven to retard the progression of ADPKD. This review focuses on the roles and mechanisms of plant-derived compounds in treating ADPKD, which may constitute promising new therapeutics in the future.

## 1 Introduction

Autosomal dominant polycystic kidney disease (ADPKD) is the most common monogenic hereditary kidney disease and the fourth leading cause of end-stage kidney disease (ESKD) (1). It affects approximately 6 million people worldwide, and approximately 50% of patients develop ESKD after 60 years of age (2). ADPKD often occurs in adults and is characterized by the development of cysts in both kidneys and an increase in total kidney volume (TKV), leading to the destruction of kidney tissue and the eventual development of renal failure, and sufferers can only be sustained by dialysis or kidney transplantation (3). Furthermore, ADPKD is a systemic disease that can cause liver cysts, pancreatic cysts, and intracranial aneurysms in addition to kidney disease, posing a severe risk to human life and health (4). Therefore, it is of great clinical significance to discover new drugs to delay the development of ADPKD.

The pathogenesis of ADPKD involves complex pathophysiological changes; mutations of the *PKD1* gene encoding polycystic protein 1 (PC1) and the *PKD2* gene encoding polycystic protein 2 (PC2) are the leading causes of ADPKD (5). In addition, ciliary dysfunction, non-antagonistic proliferation of renal tubular cells, impaired polarity of polarized and planar cells, disorder of intracellular Ca^2+^ levels, and abnormalities of cyclic adenosine monophosphate (cAMP), the mammalian target of rapamycin (mTOR), and other signaling pathways contribute to the formation and enlargement of renal cysts (6). Therefore, many drugs for treating ADPKD are primarily studied by interfering with these genetic and molecular mechanisms responsible for cystic formation.

In recent years, significant progress has been made in delaying ADPKD progression with different drugs, such as tolvaptan, rapamycin, and somatostatin. As the first drug approved by FDA for the treatment of ADPKD, tolvaptan can slow the growth of TKV and estimated glomerular filtration rate (eGFR) loss, but its hydration and potential liver injury indicate the need for further therapeutic interventions (7). In addition, rapamycin, an mTOR inhibitor, and its analogs did not show satisfactory therapeutic effects in clinical studies (8, 9).

**Figure 1:**
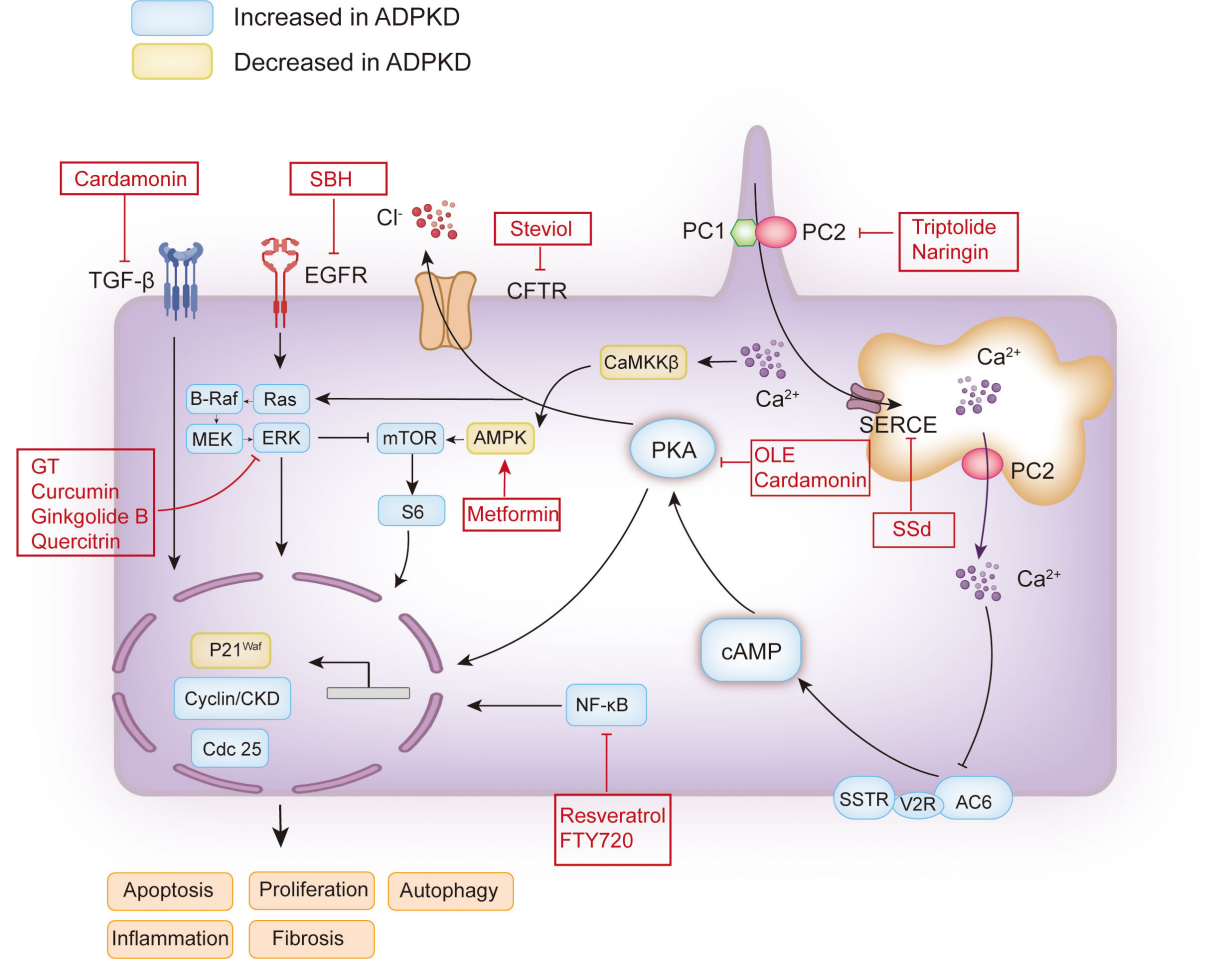
Schematic map of the pathogenesis of ADPKD and the therapeutic targets of plant extracts or plant-derived compounds. The functional site of PC1 and PC2 associated with polycystic kidney disease is on the cilia. The decrease or loss of PC1 or PC2 caused by PKD1 and PKD2 mutations may lead to a decrease in intracellular calcium concentration or an increase in intracellular cAMP. The increase of cAMP will activate PKA, which will activate the mTOR, Ras, and other signaling pathways, and promote cell proliferation. Activated PKA can also promote Cl^-^ to enter the cyst cavity through CFTR, thus promoting the secretion of cyst fluid. In addition, EGFR can promote cell proliferation by activating Ras. Additionally, calcium ions can induce autophagy by activating CaMKK β-AMPK-mTOR. The red boxes contain the candidate plant extracts or plant-derived compounds. PC1, polycystin-1; PC2, polycystin-2; AC6, adenylyl cyclase six; V2R, vasopressin type 2 receptor; SSTR, somatostatin receptor; cAMP, cyclic adenosine monophosphate; PKA, protein kinase A; CFTR, cystic fibrosis transmembrane conductance regulator; EGFR, epidermal growth factor receptor; MEK, mitogen-activated protein kinase; ERK, extracellular-signal-regulated kinase; mTOR, the mammalian target of rapamycin; SERCA, sarcoplasmic/endoplasmic reticulum Ca^2+^ ATPase; CaMKKβ, Ca^2+^/CaM-dependent protein kinase β; AMPK, AMP-activated protein kinase; TGF-β, transforming growth factor-β; SSd, saikosaponin-d; GT, *Ganoderma* triterpenes; SBH, *Sparganum stoloniferum* Buch.-Ham.

Plant-derived compounds are natural organic components, some of which are considered beneficial to health, and most are easily absorbed and metabolized in the body (10). The development of modern science and technology has broadened people’s understanding of phytochemical components. Thousands of plant-derived compounds have been used to treat various diseases, such as cancer, metabolic diseases, and neurodegeneration (11). Meanwhile, numerous plant-derived compounds have also been explored for their beneficial effects on ADPKD progression. Studies have found that several plant-derived compounds, such as saikosaponin-d, *Ganoderma* triterpenes, curcumin, ginkgolide B, steviol, resveratrol, *Sparganum stoloniferum* Buch.-Ham, *Cordyceps sinensis*, triptolide, quercitrin, naringin, cardamonin, gambogic acid, and olive leaf extract, may delay the development of cysts and improve renal function in ADPKD. Moreover, metformin, a synthesized compound derived from the French lilac or goat’s rue (*Galega officinalis*), has been proven to retard the progression of chronic kidney disease in ADPKD. This review focuses on the mechanisms (**Figure 1**) of these plant-derived compounds in treating ADPKD, which may constitute promising new therapeutics in the future.

## 2 The effect of plant-derived compounds in ADPKD models

### 2.1 Saikosaponin-d

Saikosaponin-d (SSd) is a significant triterpenoid saponin derived from *Bupleurum falcatum* L. It has immunomodulatory, anti-inflammatory, antiviral, anti-proliferation, and anti-cancer effects *in vivo* and *in vitro* (12–15). Many studies have found that SSd has an anti-tumorigenic effect, while ADPKD is considered a tumor-like disease. There are similarities between ADPKD and tumors in pathophysiology (16). This shows the application potential of SSd in the treatment of ADPKD. In ADPKD, PC1 deficiency may activate the function of sarcoplasmic/endoplasmic reticulum calcium ATPase (SERCA) and inhibit flux across the endoplasmic reticulum membrane (17). Research shows that, as a SERCA inhibitor, SSd induces autophagy *via* the direct inhibition of SERCA, which in turn upregulates intracellular calcium levels. In 2018, we reported for the first time the role of SSd in ADPKD (18). SSd directly inhibits SERCA to upregulate Ca^2+^ levels, thereby activating the CaMKKβ-AMPK-mTOR signaling pathway, which subsequently induces autophagy in ADPKD cells (18) (**Table 1**). However, this study was limited to the cellular level, and more studies are needed further to investigate the therapeutic effect of SSd in ADPKD.

**Table 1:** Summary of the function and mechanism of plant-derived compounds in ADPKD.

Compounds/ herbal medicines	Source of compound	Related signaling pathways	Effects in ADPKD	First author	Year	Country
Saikosaponin-d	*Bupleurum falcatum* L.	CaMKKβ/AMPK/mTOR	Activation of autophagy	Shi et al. (18)	2018	China
*Ganoderma* triterpenes	*Ganoderma lucidum*	RAS/MAPK	Inhibition of cell proliferation	Su et al. (19)	2017	China
Curcumin	The root of the turmeric plant	RAS/B-RAF/MEK//ERK	Inhibition of cell proliferation; promotion of cell differentiation	Gao et al. (20)	2010	China
Ginkgolide B	*Ginkgo biloba*	Ras/MAPK	Inhibition of cell proliferation	Zhou et al. (21)	2012	China
Steviol	*Stevia rebuadiana*	CFTR; APQ2	Inhibition of cell proliferation; restraint of cyst fluid secretion	Yuajit et al. (22, 23); Noitem et al. (24);	2013; 2014; 2018	Thailand
Resveratrol	Grapes, peanuts, berries, and their derivatives	NF-κB	Inhibition of inflammation	Wu et al. (25)	2016	China
*Sparganum stoloniferum* Buch.-Ham	NA	EGFR	Inhibition of cell proliferation	Xu et al. (26); Li et al. (27)	2002; 2006	China
FTY720	*Cordyceps Sinensis*	NF-κB	Inhibition of inflammation	Li et al. (28)	2019	China
Quercitrin	Vegetables and fruits	AKT/ERK	Inhibition of cell proliferation	Zhu et al. (29)	2017	China
Naringin	Flavanone naringenin and the disaccharide neohesperidose	PC2	Inhibition of cell proliferation	Waheed et al. (30)	2014	UK
Cardamonin	*Garcinia hanburyi*	MAPK/Wnt/mTOR; TGF-β/Smad2/3	Inhibition of cell proliferation; inhibition of fibrosis	He et al. (31)	2020	China
Gambogic acid	*Garcinia hanburyi*	ERK/mTOR/S6K; AMPK	Inhibition of cell proliferation	Khunpatee et al. (32)	2022	Thailand
Olive leaf extract	Olive leaf	PKA/AKT/ERK/cAMP	Inhibition of cell proliferation	Toteda et al. (33).	2018	Italy
Metformin	*Galega officinalis*	AMPK;PC2	Inhibition of cell proliferation	Takiar et al. (34); Chang et al. (35); Lian et al. (36); Perrone et al. (37)	2011; 2017; 2019; 2021	United States of America; China; China; United States of America
Triptolide	*Tripterygium wilfordii* Hook f	PC2; caspase-3	Induction of cell apoptosis; regulation of the cell cycle	Leuenroth et al. (38);	2007; 2008	United States of America

NA, not available; EGFR, epidermal growth factor receptor; MEK, mitogen-activated protein kinase; ERK, extracellular-signal-regulated kinase; AMPK, AMP-activated protein kinase; MAPK,mitogen-activated protein kinase; CFTR, cystic fibrosis transmembrane conductance regulator; mTOR, the mammalian target of rapamycin; PC2, polycystin-2; AKT, protein kinase B; TGF-β, transforming growth factor-β; NF-κB, nuclear factor-κ-gene binding; CaMKKβ, Ca^2+^/CaM-dependent protein kinase β.

### 2.2 *Ganoderma* triterpenes

*Ganoderma* triterpenes (GTs) are a family of lanostane triterpenes isolated from *Ganoderma lucidum*, which is used in Chinese traditional medicine. As primary secondary metabolites of *G. lucidum*, GTs undertake anti-cancer, anti-inflammatory, antioxidative, and hepatoprotective therapeutic activities, among others (39). They have been shown to inhibit cell proliferation and invasion, induce cell apoptosis, and regulate immune response (40). The effect of GTs on regulating multiple signaling pathways shared by ADPKD implies their possible role in modulating cyst development. In 2017, Su et al. (19) confirmed that GTs significantly downregulate the RAS/MAPK signaling pathway and inhibit renal cysts in the embryonic renal cyst model and rapidly progressive ADPKD mouse model. Further studies have shown that GT monomer CBLZ-7 (ethyl ganoderate C2) can downregulate the RAS/MAPK signaling pathway in forskolin-stimulated Madin–Darby canine kidney (MDCK) cells in a dose-dependent manner and inhibit the expansion of cysts (19). Hence, GTs have great potential to be developed as a novel therapeutic agent for treating PKD.

### 2.3 Curcumin

Curcumin is a yellow- or orange-pigmented substance obtained from the root of the turmeric plant (41). In 1937, scientific research on curcumin in the treatment of diseases was first published, and a study investigating the antibacterial activity of curcumin, published in 1949, achieved exciting results (42). More recently, it has been found that curcumin has anti-inflammatatory (43), immune regulation (44), renoprotective (45), hepatoprotective (46), and hypoglycemic (46) effects. Owing to its easy availability, low cost, and low toxicity, it is expected to be an ideal drug. Gao et al. (2011) (20) explored the mechanism of curcumin in an *in vitro* renal cyst model. The results showed that curcumin significantly inhibits the formation and enlargement of the MDCK cell cystic model and fetal renal cyst and reduced the expression of signal proteins RAS, B-RAF, p-MEK, p-ERK, c-fos, and Egr-1 in forskolin-treated MDCK cells, while increasing the expression of Raf-1 and NAB2 (20). These data suggest that curcumin may inhibit the development of renal cysts by regulating the Ras/MAPK signaling pathway and has the potential to be developed as a candidate drug for the treatment of PKD. In addition, curcumin has the disadvantage of low bioavailability and can interfere with other drugs.

### 2.4 Ginkgolide B

Ginkgolide B is a major bioactive component of *Ginkgo biloba* and undertakes anti-inflammatory (47), anti-allergy, antioxidative, anti-cancer, and neuroprotective (48–50) activities, among others. The increase of intracellular cAMP mainly stimulates the proliferation of renal cystic epithelial cells by activating the MAPK/ERK signaling pathway, which may be related to the regulation of B-Raf and Raf-1 (51, 52). Zhou et al. (21) used an *in vitro* MDCK cyst model, an embryonic kidney cyst model, and an *in vivo* PKD mouse model to study the effect of ginkgolide B on cysts. The results showed that ginkgolide B does not induce cytotoxicity and apoptosis in MDCK cells but significantly inhibits the formation and growth of renal cysts (21). Ginkgolide B could downregulate the level of B-Raf in forskolin-treated MDCK cells, but upregulate the level of Raf-1. The opposite regulation of B-Raf and Raf-1 may be the key mechanism that allows ginkgolide B to inhibit cysts (21). Therefore, the RAS/MAPK signaling pathway may be involved in the inhibitory effect of ginkgolide B on the abnormal proliferation of cystic cells.

### 2.5 Steviol

Stevioside, extracted from *Stevia rebuadiana*, is widely used as a non-calorie sweetener in food (53). Pharmacokinetic studies have shown that stevioside is first transformed into steviol, the primary metabolite, by intestinal flora, then absorbed by the intestinal tract and distributed to several organs, such as the intestine, liver, and kidneys through blood (54–56). It has been found that the interaction between steviol and renal organic anion transporter helps improve the therapeutic effect of drugs (57, 58). In addition, steviol and its derivative (dihydroisosteviol) inhibit cAMP-activated chlorine secretion by targeting CFTR in human colonic epithelial cell lines (59). The dilatation of the cyst cavity of ADPKD is related to the proliferation of cyst wall epithelial cells and the secretion of fluid. Fluid secretion depends on the chloride channel of the CFTR (60) and the water channels of the parietal membranes of the cyst wall epithelial cells (61). Chloride enters the cystic cavity through CFTR activated by cAMP, accumulates in the cyst cavity, and sucks sodium and water into the cyst cavity through the paracellular pathway, causing the cyst to enlarge (60). Yuajit et al. (2013) (22) studied the inhibitory effect of steviol and its derivatives on the cystic growth of MDCK cells and its mechanism. The results showed that steviol has the most substantial inhibitory effect on the growth of MDCK cysts, which was achieved by inhibiting the activity of the CFTR chloride channel and reducing the expression of CFTR (22). Further study in the PKD1 mouse model showed that steviol at 200mg/kg body weight for 14 days could significantly reduce kidney weight and the cystic index and improve renal function in mice. This effect is achieved in part by activating AMPK, inhibiting the expression of the CFTR chloride channel, and inhibiting the proliferation of renal epithelial cells through the mTOR/S6K pathway (23). Moreover, overexpression of aquaporin 2 (AQP2) was shown to be involved in fluid secretion, leading to cyst enlargement in ADPKD. A study in 2018 found that steviol not only affected the activity of CFTR but inhibited the expression of AQP2 at the transcriptional level and promoted the degradation of AQP2 mediated by proteasomes and lysosomes, resulting in a decrease in water transport to the cyst cavity, thus delaying the growth of cysts (24). Therefore, steviol may be a potential botanical candidate for treating PKD.

### 2.6 Resveratrol

Resveratrol is a natural polyphenol and occurs abundantly in red grapes, berries, peanuts, and legumes (62). Studies have shown that resveratrol has therapeutic effects on various diseases, such as aging, hypertension, cancer, and kidney diseases (62, 63). It has been found that resveratrol exerts its anti-inflammatory, antioxidative, and anti-proliferative effects by acting on different intracellular targets (63). Inflammation plays an essential role in the pathogenesis of ADPKD (64). Inflammatory factors have been found in the urine and renal cyst fluid of ADPKD patients (64). In addition, inflammatory cells, such as macrophages, accumulate in cystic kidneys and have been shown to promote the growth of renal cysts (64, 65). We reported the role of resveratrol in ADPKD in 2016 (25). In this study, we demonstrated that the anti-inflammatory substance resveratrol reduced the production of monocyte chemotactic protein-1, complement factor B, and tumor necrosis factor-α (TNF-α) and reduced macrophage infiltration in cystic kidneys, delaying the progression of PKD by reducing inflammation in cystic kidneys (25). Notably, resveratrol is also an activator of the SIRT pathway (66), which may have deleterious effects on PKD. However, Zhou et al. (67) found that activation of *SIRT1* led to the proliferation of renal epithelial cells through deacetylation and phosphorylation of the retinoblastoma (Rb) protein and dysregulation of cell death through deacetylation of the P53 protein, leading to continued epithelial cell growth and cystic lesion formation. Thus, excessive amounts of resveratrol may cause excessive activation of the SIRT pathway, which promotes vesicle formation and expansion. Further studies are needed to address these critical issues and identify resveratrol’s safe and effective dosage.

### 2.7 Sparganum stoloniferum Buch.-Ham

*Sparganum stoloniferum* Buch.-Ham (SBH), also known as *Coptis Chinensis*, is a commonly used traditional Chinese medicine that is widely used to improve blood circulation and reduce vascular obstruction (27). In a study, we found that SBH could prevent cells at the G0/G1 phase from reaching the G2/M phase, and inhibited the phosphorylation of EGFR, thus inhibiting the proliferation of ADPKD cystic epithelial cells (26). The *PKDL* gene encodes polycystic protein-L (PCL), which has 50% homology with PC2 (68). PC2 and PCL are non-selective cation channels for the permeability of potassium, sodium, and calcium, which are closely related to the occurrence of renal cysts (69). Li et al. (27) expressed human PCL in Xenopus oocytes and examined the effects of SBH on PCL channel function, using the 2-electrode voltage-clamp technique and radiolabeled ^45^Ca uptake measurements. The results showed that SBH contained one or more components that inhibited PCL channels, which might be useful for diseases related to abnormal PCL function (27). However, whether SBH also inhibits PC2 channels remains to be determined.

### 2.8 Cordyceps sinensis

*Cordyceps Sinensis* (CS) is a unique leafy fungus growing on caterpillars and is regarded as a beneficial herbal medicine in traditional Chinese medicine and is used to treat many diseases, including those that affect the respiratory system, liver, cardiovascular system, as well as hyperlipidemia (70). CS has renal protective effects (71) and has been used to treat several renal diseases, including chronic renal failure, renal transplantation, and acute renal injury (72–74). FTY720 (Fingolimod) is a novel immunomodulatory compound derived from CS and is an effective inhibitor of sphingosine-1-phosphate receptor (S1PR) (75). S1P has been approved by the FDA for the treatment of multiple sclerosis. S1P is an inflammatory regulator that activates the signal transducer and activator of the transcription 3 (STAT3) pathway or directly activates the NF-κB pathway through S1PR1 (76). We found that FTY720 can inhibit the expression of pro-inflammatory cytokines, such as IL-6 and tumor necrosis factor-α(TNF-α), block the activation of inflammatory pathways, such as STAT and NF-κB, and thus inhibit the growth of renal cysts in PKD rats (28).

### 2.9 Quercitrin

Quercitrin is a kind of plant polyphenol widely found in many kinds of vegetables and fruits (77). It has many pharmacological effects, such as those that are anti-inflammatory, anti-tumorigenic, anti-oxidative, neuroprotective, and anti-aging (78–80). Studies have shown that quercitrin significantly inhibits the growth and proliferation of tumor cells through MAPK/ERK and AKT/mTOR (81, 82), which corresponds to the pathophysiology of ADPKD. Zhu et al. (29) used an MDCK cystic model and a PKD mouse model to study the effect of quercitrin on renal cysts. The results showed that quercitrin significantly inhibits the formation and development of vesicles both *in vivo* and *in vitro* in a dose-dependent manner (29). Quercitrin significantly decreases the levels of AKT and ERK in the kidney cells of PKD mice. In addition, E-cadherin is a kind of cell membrane protein that is involved in the maintenance of intercellular adhesion and plane polarity (83, 84) and can be regulated through the regulation of the ERK and AKT signal pathways (85). Zhu et al. showed that the expression of E-cadherin is reduced in PKD mice and is located in proximal tubules (29). Quercitrin reversed E-cadherin expression in proximal tubules of PKD mice on P10. Meanwhile, quercitrin can decrease p-ERK and p-AKT expression, thus inhibiting cystic development (29). Therefore, quercitrin has great development potential as a candidate drug for treating ADPKD.

### 2.10 Naringin

Naringin is a flavanone compound found in citrus fruits and has anti-inflammatory, antioxidative, and cholesterol-lowering effects (86). In addition, naringin can promote cell cycle arrest and p53-dependent apoptosis to inhibit the growth of tumor cells (87). Waheed et al. (30) studied the effect of naringin on the growth of cysts in PC2 knockout MDCK cells. The results showed that naringin can significantly reduce the viability and inhibit the growth of MDCK cells in a PC2-dependent manner (30). As PC2 participates in intracellular calcium signal transduction, thus affecting the secretion of Cl^-^, the effect of naringin on Cl^-^ was investigated (30). However, naringin did not reduce the short-circuit current compared with the positive control group (e.g., genistein and apigenin), which inhibited the entry of chloride ions through the basement membrane (30). This suggests that naringin exerts its anti-proliferative effect by activating PC2, but the downstream mechanism does not include liquid secretion (30).

### 2.11 Cardamonin

Cardamonin is a chalcone compound isolated from *Alpinia katsumadai* and which has significant anti-inflammatory, anti-proliferative, and immunomodulatory effects (88). Many studies have shown that cardamonin has an obvious curative effect on many diseases, such as arthritis (89), colitis (90), and cancer (91). It has been found that the occurrence of ADPKD is related to the changes in the composition of the extracellular matrix and the thickness of the basement membrane. The excessive production of collagen leads to the deposition of fibroblasts and collagen fibers (92). He et al. (31) studied the role of cardamonin in the treatment of a cyst enlargement MDCK cyst model, an embryonic kidney cyst model, and an orthologous mouse model of ADPKD. The results showed that cardamonin delayed cystic growth and alleviated renal fibrosis by downregulating the MAPK, Wnt, mTOR, and TGF-β signaling pathways (31).

### 2.12 Gambogic acid

Gambogic acid (GA) is a compound isolated from the brownish orange gamboge resin of the *Garcinia hanburyi* tree (93) and undertakes anti-proliferative, anti-apoptotic, anti-tumorigenic, anti-angiogenic, and anti-inflammatory activities, among others (93–98). GA has been shown to inhibit the proliferation of melanoma (95), esophageal squamous carcinoma (99), and glioma cells (100). Khunpatee et al. (32) studied the effect of GA on ADPKD in MDCK and PKD1 mutant cells. The results showed that GA inhibits the enlargement of cysts by inhibiting the phosphorylation of the ERK1/2 and mTOR/S6K signaling pathways (32). In addition, GA can significantly improve the phosphorylation activity of AMPK (32).

### 2.13 Olive leaf extract

Extra virgin olive oil contains a lot of polyphenols, which can lower blood pressure, increase blood flow in coronary arteries, and slow down heart rate (101, 102). Olive leaf extract (OLE) is often used to prevent and treat high blood pressure or as a diuretic or preservative (103). Recent studies have found that OLE can treat a variety of cancers (104–107). Toteda et al. (33) investigated whether OLE can inhibit the cystic growth of ADPKD *in vitro*. The results showed that OLE could reduce the level of PKA, p-ERK, and cAMP and upregulate the level of p-AKT, thus reducing the growth of cystic cells *in vitro* (33).

## 3 The effect and safety of plant-derived compounds in ADPKD patients

### 3.1 Metformin

Metformin is a synthesized compound derived from goat’s rue (*Galega officinalis*). As an AMPK activator, metformin has been widely used for treating type 2 diabetes and polycystic ovary syndrome for decades (108). During the development of PKD, AMPK activity decreases. Several studies have shown that AMPK is a potential target for treating ADPKD (34). In preclinical studies, metformin can inhibit renal cysts in ADPKD mice, miniature pig models, and *in vitro* experiments. Therapeutic AMPK activation can reduce the severity of cystic kidney disease in *Pkd^-/-^* animal models by improving mitochondrial biogenesis and reducing tissue inflammation (109). Recent preclinical studies have shown that it may play a role in delaying the development of renal cysts in patients with ADPKD (110). The therapeutic effect of metformin on ADPKD was first proposed by Takiar et al. (34). They found that large doses (300 mg/kg body weight) of metformin can stimulate AMPK, resulting in the inhibition of CFTR and mTOR, thereby inhibiting the secretion and proliferation of *Pkd^-/-^* mouse epithelial cells (34). Another study showed that metformin inhibits the formation of renal cysts in *Pkd2* morphant zebrafish by activating the AMPK pathway and reducing cell proliferation and autophagy (35). Similarly, in a miniature *Pkd^-/-^* pig model, oral metformin can inhibit renal cyst growth and improve renal function (36). However, no beneficial effect of metformin was observed in another *Pkd^-/-^* mouse model (111). The reason for this may be that this study injected tamoxifen later causing the disease to progress slowly compared with the study by Takiar et al. Another difference between the two studies may be the method of drug administration. Takiar et al. administered the drug by injection (34), while Leonhard et al. (111) administered it orally. The oral bioavailability of metformin was only 40–60%. This suggests that the animal models and administration methods used in the study will affect metformin’s effectiveness. Furthermore, the tolerance, safety, and preliminary efficacy of metformin in adult patients with ADPKD were evaluated in a phase 2 double-blind placebo-controlled randomized controlled trial (37). In this study, 97 ADPKD patients between the ages of 18 and 60 were randomly assigned to receive a 1:1 administration of metformin and placebo. The results showed that metformin slightly reduces the decrease of GFR in patients with ADPKD, but the effect was not significant (37). Encouragingly, metformin showed good safety and tolerance in this study (37). Hence, the evaluation of efficacy requires a larger trial with sufficient power to detect differences in endpoints. Interestingly, the phase 3 clinical trial (IMPEDE-PKD) of metformin therapy to alleviate renal function decline in ADPKD is expected to be completed in 2026. By then, we should have a clear answer as to whether metformin will be effective in the management of ADPKD.

### 3.2 Triptolide

Triptolide is a compound derived from the Chinese herbal medicine *Tripterygium wilfordii* Hook f (TWHf) and exhibits anti-proliferative, immunosuppressive, and anti-inflammatory effects in many diseases (112). Root extracts of TWHf have been used to treat nephrotic syndrome, cancer, lupus, Behçet’s disease, and other diseases throughout history (113, 114). Since triptolide was extracted and isolated from TWHf in 1972, its mechanism of action and clinical efficacy have been extensively investigated, and its inhibitory effect on renal cysts has also become a research hotspot. Several studies have shown the inhibitory effect of triptolide on renal cysts in several premature *Pkd1* animal models at embryogenic, neonatal, or neonatal to adult transition stages (38, 115, 116). Research shows that triptolide can regulate the release of Ca^2+^ through a PC2-dependent mechanism to arrest cyst proliferation (116). In 2018, we reported the effect of triptolide in an adult PKD rat model. Triptolide treatment for 12 weeks delayed the decline of renal function and inhibited renal cysts in adult PKD rats, perhaps through the JAK2/STAT3 pathway (117). In a clinical study, Chen et al. found that albuminuria decreases in patients with ADPKD after 6 months of triptolide treatment, and cyst growth rate and renal dysfunction are significantly improved (118). However, the study also mentions that the use of triptolide in treating ADPKD may cause menstrual disorders in female patients (118), and the water solubility of triptolide is poor (118). Hence, we need more rigorous pharmacological research and well-designed clinical trials to provide evidence-based support for its safety and efficacy.

## 4 Conclusions and outlook

ADPKD patients show extreme enlargement of both kidneys, which are filled with cystic fluid, and this eventually leads to ESKD (2). At present, the limited treatment options for ADPKD is still a challenge for nephrologists. So far, some plant-derived compounds have been shown to inhibit the activity of renal cysts through a variety of potential mechanisms, which provides new options for ADPKD treatment. However, there are still some problems that need to be considered in the future development of plant-derived compounds. First, some plant-derived compounds inhibit renal cysts *in vitro* but much less so *in vivo* or in clinical trials, which may be due to the toxicity or low bioavailability of the drugs. Therefore, more basic and clinical studies are needed to prove the safety and effectiveness of these plant-derived compounds. Second, there are many factors affecting the effectiveness of plant-derived compounds, and good quality control is the key to ensuring their safety and effectiveness. Therefore, a more comprehensive quality control model is needed, which requires pharmacological/biological evaluation in addition to some emerging chemical analysis assessments, such as chromatographic fingerprinting and multi-component quantification (119, 120). Besides, the nephrotoxicity of plant-derived compounds cannot be ignored (121). In addition to aristolochic acid, some other plant-derived compounds, such as *Tripterygium regelii* Sprague et Takeda, can cause renal tubular damage and inflammatory cell infiltration (122). It is speculated that more than 100 herbs have adverse effects on the kidneys (123). In addition, although many plant-derived compounds have been widely used in clinical environments, the mechanism of most plant-derived compounds is still unclear. A more comprehensive understanding of the specific mechanisms will lead to the discovery of more potent drugs to treat ADPKD. Finally, the application of artificial intelligence technology and the development of bioinformatics may provide new insights for research of plant-derived compounds for treating ADPKD. In addition to those described in this paper, there are some other plant-derived compounds, such as colchicine and emodin, that are associated with the pathophysiology of ADPKD (124, 125).

In conclusion, plant-derived compounds provide a rich resource for the development of drugs in the treatment of ADPKD. Many plant-derived compounds show good application potential for the inhibition of renal cyst growth and may provide promising new therapeutic choices for ADPKD in the future.

## Author contributions

All authors listed have made a substantial, direct, and intellectual contribution to the work and approved the manuscript for publication.
